# The Public’s Knowledge of Osteoarthritis and Its Related Risk Factors in Makkah, Saudi Arabia

**DOI:** 10.7759/cureus.35457

**Published:** 2023-02-25

**Authors:** Mohammed K Alghamdi, ‌‏Mohamed A Elhefny, Khalid A Basamih, Maria A AlSulami, Nawaf O Amodi, Eyad J Algahwaji, Azzam A Hanif, Aryaf F Alobaidi, Hussam A Alraddadi, Sayyaf M Alhazmi

**Affiliations:** 1 Department of Medicine and Surgery, College of Medicine, Umm Al-Qura University, Makkah, SAU; 2 Department of Medical Genetics, College of Medicine, Umm Al-Qura University, Al Qunfudhah, SAU

**Keywords:** inflammatory disease, joint stiffness, joint destruction, loss of mobility, chronic progressive disease, musculoskeletal disorder

## Abstract

Background

Osteoarthritis (OA) is a chronic progressive disease that leads to the destruction of the articular cartilage inside the joint. OA is a common everyday musculoskeletal disorder worldwide, and it is believed that OA is triggered by genetics and environmental factors, including age, which is the most significant risk factor. This study aimed to investigate the general population’s knowledge of OA and its related risk factors in Makkah, Saudi Arabia.

Methodology

This cross-sectional study was performed between December 2022 and January 2023 among the general population of Makkah, Saudi Arabia using an online survey using Google Forms. An appropriate statistical analysis was then conducted on the collected data.

Results

A total of 1,087 participants enrolled in this study. According to the multivariate logistic regression analysis, 48% (n = 789) of the participants reported that OA occurs due to joint cartilage age and use. In total, 69.7% of the participants knew that OA is a chronic problem, 84.4% knew it is a common disease, and 39.3% thought that all types of joints can suffer from OA. Over half (53.1%) of the participants knew that joint stiffness is a sign of OA, and 63.4% thought that OA may lead to the loss of joint motion. Over four-fifths (82.5%) thought that advancing age is a risk factor for OA, and 27.5% incorrectly thought that the incidence of OA is equal between men and women. Overall, 62.9% of the participants knew about clinical examinations and X-rays. Moreover, 78% thought that physiotherapy can improve the symptoms of OA, and 65.3% thought that some types of exercise can help. Finally, 35.8% of the participants had an overall awareness of OA, while 64.2% had poor awareness.

Conclusions

The general public of Makkah showed low knowledge of OA and its associated risk factors. Many misunderstandings about the causes, risk factors, and treatment of OA were acknowledged. Awareness campaigns with brochures and flyers can be used to raise the population’s knowledge.

## Introduction

Osteoarthritis (OA) is a chronic progressive disease caused by inflammatory and metabolic factors that lead to the destruction of the joint’s articular cartilage, resulting in stiffness and swelling, a loss of mobility, and pain (the most prominent symptom among patients with OA) [1]. OA is the most prevalent musculoskeletal disorder worldwide and is believed to be caused by genetics and environmental factors [2-4]. The most significant risk factors for OA of all joints include age, which is the greatest risk factor, hormonal changes, and obesity as it leads to high stress on the joints and cartilage [5-7]. Moreover, the most prevalent kind of joint disease in the United States is OA [5]. The prevalence and incidence rates of OA differ among studies globally, with approximately 240 million individuals worldwide suffering from OA [8]. The prevalence has increased from 21 million in 1990 to 27 million in 2005, with over 30 million in the United States [6]. Only a few studies have been conducted on OA among medical students in certain cities in Saudi Arabia. One such study was conducted among the public population in the Asser region [9]. However, it focused on knee OA and limited its data collection methods to those who could read. Studies have recommended that more attention be paid to improving the awareness of OA consequences, especially among young people [10]. In addition, a study of Saudi adults in Tabuk was limited by its sample of participants and their relatives and friends recruited through snowball sampling [11]. Another study of the public population in Jeddah measured knowledge of OA, and the findings showed low levels of knowledge of OA and the risk factors connected to it [12]. However, no study has thus far investigated the knowledge among the public in Makkah. To address this issue, we conducted an online survey to investigate the general population’s understanding of OA and consider the limitations of previous studies.

## Materials and methods

Study design and participants

This cross-sectional study included participants from the general public in Makkah, Saudi Arabia. All participants were above 18 years of age, and both men and women were included in the study.

Ethical considerations and sample size

A self-administrated online questionnaire was distributed between December 2022 and January 2023 via social media platforms to collect data after obtaining ethical approval from the Biomedical Ethics Committee of the College of Medicine Committee at Umm Al-Qura University, Makkah, Saudi Arabia (approval number: HAPO-02-K-012-2022-11-1357). OpenEpi version 3.0 was used to calculate the sample size of this study, keeping the confidence interval at 95% and considering a 50% prevalence of the sample size [13]. The minimal recommended sample size was 384 participants. However, we included more than 500 participants to increase the generalizability and accuracy of the results.

Study tool

This study adapted and translated a validated assessment tool [12]. After a group of expert researchers had translated the questionnaire into Arabic, the self-assignment questionnaire, including three sections in Arabic, was organized. In the first section, demographic data were collected, such as sex, age, and education level. In the next section, we questioned the participants about whether they understood the OA definition. The third section assessed the participants’ knowledge.

Statistical analysis

The data were collected, reviewed, and fed to SPSS version 21 (IBM Corp., Armonk, NY, USA). All statistical methods used were two-tailed with an alpha level of 0.05. Significance was considered at p-values of less than or equal to 0.05. Regarding awareness, each correct answer was given one point. The overall awareness of OA was assessed by summing the discrete scores for the different correct awareness items. The total awareness score was categorized as poor if the participants’ score was less than 60% of the total score, while a good level of awareness was considered if the participants’ score was 60% or more. A descriptive analysis was performed based on the frequency distribution and percentage of the study variables, including the participants’ personal data and if they knew someone with OA. Further, the participants’ awareness of OA was tabulated, while the overall awareness level and source of information were graphed. A cross-tabulation was used to show the distribution of the participants’ overall awareness level based on their personal data and other factors. We used the Pearson chi-square test for significant values and the exact probability test if there were small frequency distributions.

## Results

A total of 1,638 participants meeting the inclusion criteria completed the study questionnaire. The participants’ ages ranged from 18 to 69 years, with a mean age of 26.5 ± 14.9 years. Altogether, 1,012 (61.8%) participants were women and 1,262 (77%) had a university level of education or above, while 44 (2.7%) had below a secondary level of education. A total of 1,039 (63.4%) participants knew someone diagnosed with OA (Table 1).

**Table 1:** Personal data of study participants, Makkah city, Saudi Arabia.

Personal data	Number	%
Age in years		
18–29	834	50.9%
30–39	185	11.3%
40–49	303	18.5%
50–59	249	15.2%
60+	67	4.1%
Gender		
Male	626	38.2%
Female	1,012	61.8%
Educational level		
Below secondary	44	2.7%
Secondary	332	20.3%
University/above	1,262	77.0%
Do you know someone diagnosed with osteoarthritis?		
Yes	1,039	63.4%
No	399	24.4%
Not sure	200	12.2%

**Figure 1:**
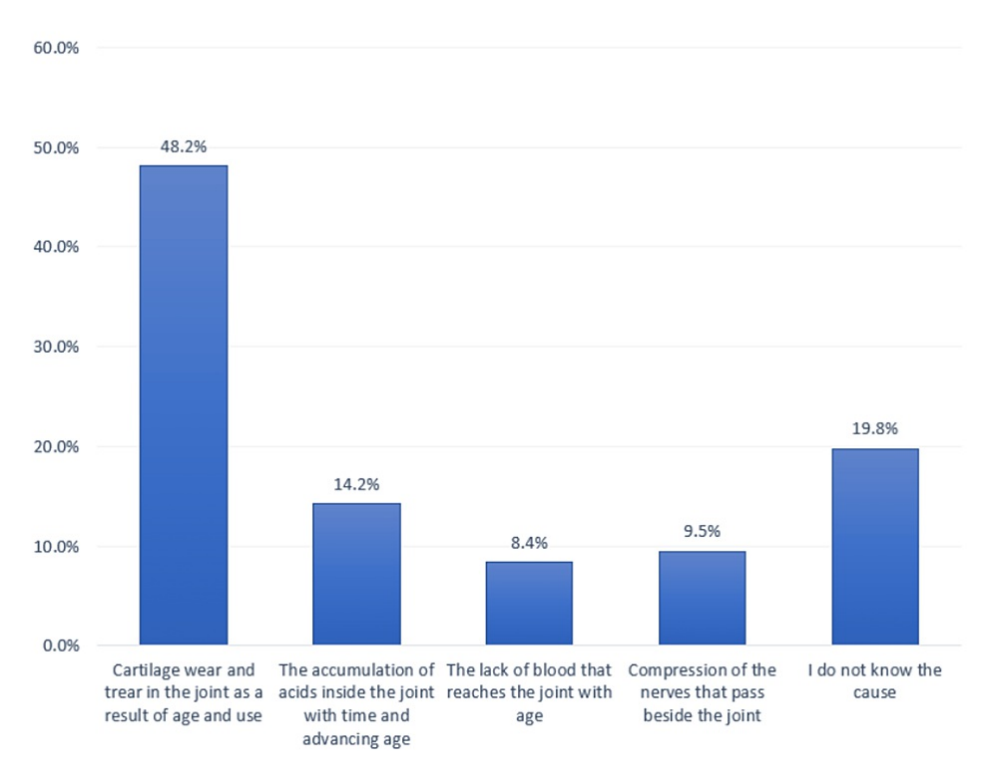
Participants’ awareness regarding the mechanism of osteoarthritis development.

In total, 789 (48.2%) participants reported that OA occurs due to cartilage wear and tear in the joint as a result of age and use (Figure 1).

Generally, 69.7% of the participants understood that OA is a long-standing problem, 84.4% knew that it is a common disease, and 39.3% thought that all types of joints may suffer from OA. Regarding signs and symptoms, 53.1% knew that joint stiffness is a sign of OA, 40.5% reported swelling, and 63.4% thought that OA may lead to the loss of joint motion. Regarding risk factors, 82.5% thought that advancing age is a risk factor for OA, 33.5% thought that genetics are a risk factor for OA, and 27.5% incorrectly thought that the incidence of OA is equal between men and women. Regarding the diagnosis of OA, 62.9% knew about clinical examination and X-rays, while 45.6% stated that blood tests are not used to diagnose OA. Considering the treatment of OA, 78% thought that physiotherapy can improve the manifestation of OA, 65.3% thought that some types of work-out, such as swimming, are suitable for OA patients, 35% thought that analgesics may improve symptoms, and 37.3% thought that joint replacement procedures are the best and last solution for OA (Table 2).

**Table 2:** Community awareness about osteoarthritis and its related risk factors in Makkah city, Saudi Arabia.

Domain	Items	Yes		No		Not sure	
		Number	%	Number	%	Not sure	%
General awareness	Do you think that osteoarthritis is a chronic problem?	1,142	69.7%	256	15.6%	240	14.7%
	Do you think that osteoarthritis is rare?	118	7.2%	1,382	84.4%	138	8.4%
	Do you think that all types of joints may suffer from osteoarthritis?	644	39.3%	584	35.7%	410	25.0%
	Do you think that osteoarthritis is the result of a cold or moist atmosphere?	314	19.2%	890	54.3%	434	26.5%
	Do you think that osteoarthritis is caused by bacteria?	202	12.3%	951	58.1%	485	29.6%
Signs & symptoms	Do you think that pain is the only symptom of osteoarthritis?	472	28.8%	925	56.5%	241	14.7%
	Do you think joint stiffness is a sign of osteoarthritis?	870	53.1%	260	15.9%	508	31.0%
	Do you think swelling is a sign of osteoarthritis?	663	40.5%	492	30.0%	483	29.5%
	Do you think that osteoarthritis may lead to loss of joint movement?	1,039	63.4%	252	15.4%	347	21.2%
Risk factors	Do you think that genetics is a risk factor for osteoarthritis?	548	33.5%	626	38.2%	464	28.3%
	Do you think that advancing age is a risk factor for osteoarthritis?	1,351	82.5%	146	8.9%	141	8.6%
	Do you think that the incidence of osteoarthritis is equal between males and females?	451	27.5%	800	48.8%	387	23.6%
Diagnosis	Do you think that clinical examination and X-rays are used to diagnose osteoarthritis?	1,031	62.9%	178	10.9%	429	26.2%
	Do you think that a blood test is used to diagnose osteoarthritis?	350	21.4%	747	45.6%	541	33.0%
Management	Do you think that analgesics may improve symptoms?	574	35.0%	741	45.2%	323	19.7%
	Do you think that some types of exercises, such as swimming, are suitable for osteoarthritis patients?	1,069	65.3%	201	12.3%	368	22.5%
	Do you think the acid-free diet improves osteoarthritis?	448	27.4%	215	13.1%	975	59.5%
	Do you think that physiotherapy can improve the symptoms of osteoarthritis?	1,278	78.0%	120	7.3%	240	14.7%
	Do you think that oil needles or stem cell needles are a definitive treatment for osteoarthritis?	348	21.2%	546	33.3%	744	45.4%
	Do you think that joint replacement surgery is the best and last solution for osteoarthritis?	611	37.3%	483	29.5%	544	33.2%

**Figure 2:**
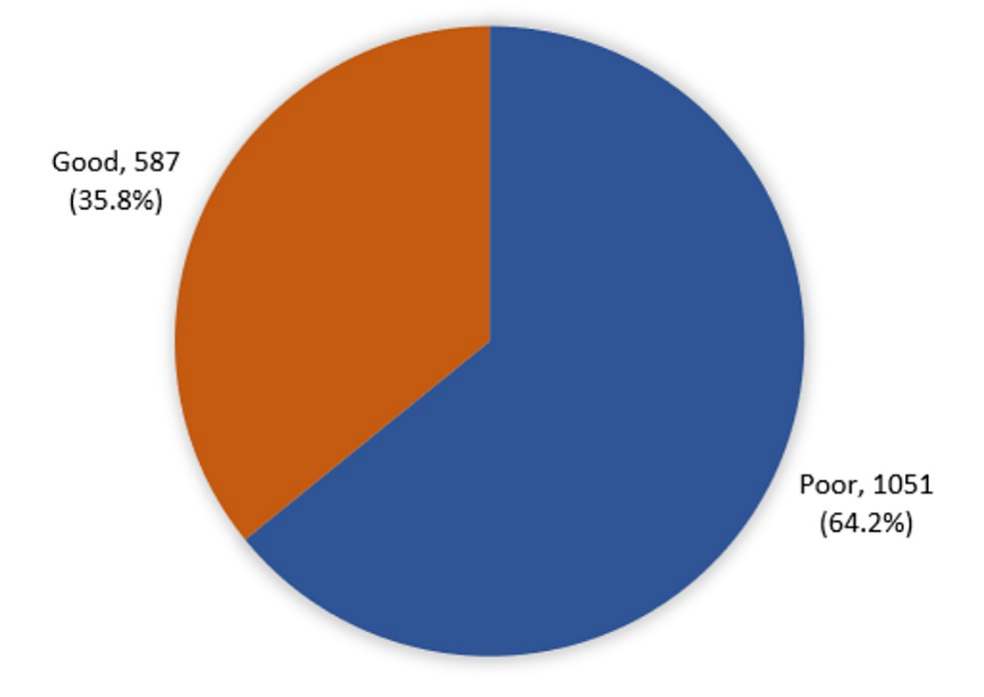
Overall community awareness level about osteoarthritis and its related risk factors in Makkah city, Saudi Arabia.

A total of 587 (35.8%) participants had an overall awareness level regarding OA while 1,051 (64.2%) had poor awareness levels (Figure 2).

**Figure 3:**
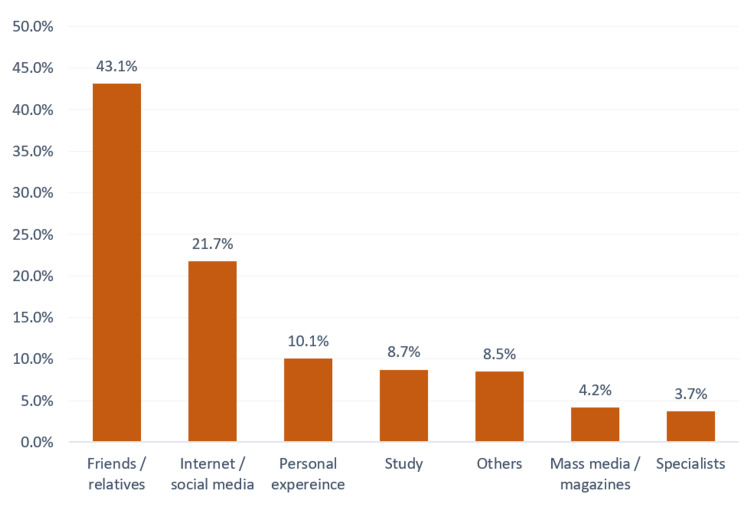
Sources of information about osteoarthritis and its related risk factors in Makkah city, Saudi Arabia.

The most reported source was friends and family (43.1%), followed by the internet and social media (21.7%), personal experience (10.1%), and one’s studies (8.7%) (Figure 3).

In total, 40% of the participants who knew someone diagnosed with joint roughness had a good awareness level compared with 30.8% of those who did not (p = 0.001). Moreover, 49% of the participants who sourced their information from their studies had a good awareness level of OA compared with 41% who reported specialists and 21.6% who reported others (p = 0.001) (Table 3).

**Table 3:** Factors associated with participants’ awareness regarding osteoarthritis.

Factors	Awareness level				P-value
	Poor		Good		
	Number	%	Number	%	
Age in years					0.176
18–29	544	65.2%	290	34.8%	
30–39	119	64.3%	66	35.7%	
40–49	181	59.7%	122	40.3%	
50–59	157	63.1%	92	36.9%	
60+	50	74.6%	17	25.4%	
Gender					0.416
Male	394	62.9%	232	37.1%	
Female	657	64.9%	355	35.1%	
Educational level					0.895
Below secondary	29	65.9%	15	34.1%	
Secondary	216	65.1%	116	34.9%	
University/above	806	63.9%	456	36.1%	
Do you know someone diagnosed with joint roughness?					0.001
Yes	623	60.0%	416	40.0%	
No	276	69.2%	123	30.8%	
Not sure	152	76.0%	48	24.0%	
Source of information					0.001
Specialists	36	59.0%	25	41.0%	
Friends/relatives	470	66.6%	236	33.4%	
Internet/social media	230	64.6%	126	35.4%	
Personal experience	89	53.9%	76	46.1%	
Study	73	51.0%	70	49.0%	
Mass media/magazines	44	64.7%	24	35.3%	
Others	109	78.4%	30	21.6%	

## Discussion

This study investigated the knowledge of OA in the public population of Makkah. Of the 1,638 participants, more than half had insufficient knowledge, in line with multiple studies conducted in Tabuk and Jeddah [11,12]. Interestingly, two studies conducted in Qunfudah and Aseer reported a higher level of knowledge than those in our study [9,14]. The knowledge and awareness of OA differ across Saudi Arabia, with similar findings found in our study and in the study in Jeddah. This can be explained by the close distance between the two cities and the population sharing a common cultural background [12]. In this study, half of the participants were aged 18-29 years; furthermore, most respondents were women. Similarly, 43.1% were aged 18-24 years in the Tabuk study, and most respondents were women (85.5%) [11]. The study in Jeddah reported more female respondents than male respondents, with half of the respondents aged 18-29 years [12]. These findings are contrary to the study in Aseer, whose sample was predominantly aged 30-49 years and included more men than women [9]. The younger population has insufficient knowledge and awareness of OA compared with older age groups, which may be expected, as the disease is more common in older age groups.

Limitations

There was an unequal sex distribution as more women responded to the survey than men. Further, half of the participants were aged between 18 and 29 years (50.9%), which suggests a biased selection. Despite the size of the study sample, it only represented a single Saudi Arabian city. Consequently, this may have an effect on the generalisability of the findings to other regions of Saudi Arabia.

## Conclusions

This study showed a low knowledge of OA and its related risk factors. It is an avoidable disease that affects patients and their quality of life. Many misunderstandings about OA were acknowledged; hence, enhancing the public’s understanding would add notable value. Awareness campaigns with brochures and flyers can also raise the population’s knowledge, resulting in the early recognition of the disease, fixing misunderstandings, and controlling dangerous and unproven procedures in OA management. More studies are needed across the region to investigate the population’s knowledge and provide doctors with proof of familiar misconceptions.
